# LLIN Evaluation in Uganda Project (LLINEUP): modelling the impact of COVID-19-related disruptions on delivery of long-lasting insecticidal nets on malaria indicators in Uganda

**DOI:** 10.1186/s12936-024-05008-8

**Published:** 2024-06-06

**Authors:** Jaffer Okiring, Samuel Gonahasa, Catherine Maiteki-Sebuguzi, Agaba Katureebe, Irene Bagala, Peter Mutungi, Simon P. Kigozi, Jane F. Namuganga, Joaniter I. Nankabirwa, Moses R. Kamya, Martin J. Donnelly, Thomas S. Churcher, Sarah G. Staedke, Ellie Sherrard-Smith

**Affiliations:** 1 Clinical Epidemiology Unit, Makerere University College of Health Sciences, PO Box 7475, Kampala, Uganda; 2 Infectious Diseases Research Collaboration, Kampala, Uganda; 3 Department of Medicine, Makerere University, Kampala, Uganda; 4 Department of Vector Biology, Liverpool School of Tropical Medicine, Liverpool, UK; 5 Wellcome Sanger Institute, Hinxton, UK; 6 MRC Centre for Global Infectious Disease Analysis, Imperial College London, London, UK; 7 Department of Clinical Research, London School of Hygiene & Tropical Medicine, London, UK

**Keywords:** Malaria, Modelling, COVID-19, Uganda, Long-lasting insecticidal nets

## Abstract

**Background:**

Disruptions in malaria control due to COVID-19 mitigation measures were predicted to increase malaria morbidity and mortality in Africa substantially. In Uganda, long-lasting insecticidal nets (LLINs) are distributed nationwide every 3–4 years, but the 2020–2021 campaign was altered because of COVID-19 restrictions so that the timing of delivery of new nets was different from the original plans made by the National Malaria Control Programme.

**Methods:**

A transmission dynamics modelling exercise was conducted to explore how the altered delivery of LLINs in 2020–2021 impacted malaria burden in Uganda. Data were available on the planned LLIN distribution schedule for 2020–2021, and the actual delivery. The transmission model was used to simulate 100 health sub-districts, and parameterized to match understanding of local mosquito bionomics, net use estimates, and seasonal patterns based on data collected in 2017–2019 during a cluster-randomized trial (LLINEUP). Two scenarios were compared; simulated LLIN distributions matching the actual delivery schedule, and a comparable scenario simulating LLIN distributions as originally planned. Model parameters were otherwise matched between simulations.

**Results:**

Approximately 70% of the study population received LLINs later than scheduled in 2020–2021, although some areas received LLINs earlier than planned. The model indicates that malaria incidence in 2020 was substantially higher in areas that received LLINs late. In some areas, early distribution of LLINs appeared less effective than the original distribution schedule, possibly due to attrition of LLINs prior to transmission peaks, and waning LLIN efficacy after distribution. On average, the model simulations predicted broadly similar overall mean malaria incidence in 2021 and 2022. After accounting for differences in cluster population size and LLIN distribution dates, no substantial increase in malaria burden was detected.

**Conclusions:**

The model results suggest that the disruptions in the 2020–2021 LLIN distribution campaign in Uganda did not substantially increase malaria burden in the study areas.

**Supplementary Information:**

The online version contains supplementary material available at 10.1186/s12936-024-05008-8.

## Background

Malaria remains a major public health problem, particularly in Africa [1]. Malaria control efforts have been challenged by insufficient funding, the emerging threats of anti-malarial drug and insecticide resistance [2–5], and more recently, the COVID-19 pandemic. Disruptions in malaria control activities, including delays in distribution of long-lasting insecticidal nets (LLINs), due to the COVID-19 pandemic were predicted to substantially increase malaria morbidity and mortality [6, 7]. Decreased availability of anti-malarial medications and rapid diagnostic tests due to interrupted supply chains [8, 9], and limited access to health facilities due to national lockdowns and travel restrictions [10, 11], were also raised as potential concerns. The World Health Organization (WHO) and others predicted that if LLIN distribution was halted and malaria case management was significantly disrupted, malaria deaths in sub-Saharan Africa could double compared to 2018 [12, 13]. However, little evidence is available on the impact of the COVID-19 restrictions on malaria burden. The LLINEUP trial in Uganda provided a unique opportunity to evaluate the impact of changes in the LLIN distribution timelines due to the pandemic on malaria indicators in Uganda.

LLINs are the cornerstone of malaria control in many African countries, including Uganda [14]. To achieve universal coverage, the WHO recommends delivering LLINs free-of-charge through nationwide mass distribution campaigns every 3 years [15]. Uganda’s Ministry of Health and supporting partners have conducted three national ‘universal coverage campaigns’ to deliver LLINs (in 2013–2014, 2017–2018, and 2020–2021). In Uganda, the Ministry of Health (MoH) collected household registration data to determine the appropriate number of LLINs to deliver to each household aiming to cover two people by each net delivered, rounding up if an uneven number of people reside in a household [14, 16]. The most recent campaign coincided with the COVID-19 pandemic [17] and was disrupted by restrictions imposed by the Ugandan government, to restrict the spread of COVID-19. Although the Ugandan MoH endeavoured to deliver LLINs according to the original timelines, the COVID-19 restrictions led to alterations in the distribution schedule transmission with delivery delayed in some areas [11] and accelerated in others.

Transmission modelling can allow us to consider scenarios that cannot be tested in reality, and compare these to what actually happened, to estimate the impacts of various events. In this case, an established and freely available transmission model (malaria simulation, [18]) was employed to explore how the altered timing of LLIN delivery may have interrupted malaria control in Uganda. After making explicit assumptions, and maintaining parameters between scenarios with the exception of the timing of LLIN delivery, this specific impact of the COVID-19 induced challenges can be quantified. The LLINEUP study was a cluster-randomized trial embedded into Uganda’s 2017–2018 LLIN distribution campaign that collected data from 2017 to 2019 [16, 19]. This provides understanding of context for 100 distinct health sub-districts to help calibrate the transmission model simulations. In each health sub-district that was used as a cluster for the trial, model parameters were matched to reflect baseline pyrethroid resistance profiles of local mosquitoes, their species composition and bionomics, the initial net use across the community, how net use waned over time after receiving a new net, and assumed seasonal dynamics of the region. Uncertainty across these parameter estimates was generated using the trial data and understanding of LLIN performance in the context of pyrethroid resistant mosquitoes from previous work [20, 21]. This work explored how COVID-19 mitigation measures may have influenced malaria burden in Uganda, given the altered delivery of LLINs during the 2020–2021 campaign.

## Methods

### LLINEUP trial

A total of 104 health sub-districts (clusters) in eastern and western Uganda were included in the LLINEUP trial, covering 48 of 121 (40%) districts [19]. Entomological and epidemiological data, collected in the LLINEUP trial from 2017 and 2019, were used to calibrate model simulations in 100 of the original 104 clusters; in three clusters multiple LLINs were distributed. Because no dominant net type was received, these clusters were dropped prior to the analysis [16, 22]. One cluster (in Budiope) was dropped because pyrethroid-pyriproxyfen nets were distributed in this region and these nets are yet to be characterized within the existing modelling framework. Data were available on the planned LLIN distribution schedule for 2020–2021, and when LLINs were actually distributed. Clusters were further divided into sub-counties for LLIN distribution, but it was not possible to parameterize parasite prevalence or vector density estimates, nor estimate population sizes, for all regions at this finer scale. Instead, the study aimed to simulate the impact, at the cluster level, of delivering the most abundant net-type during the 2020–2021 campaign, including LLINs with pyrethroid-piperonyl butoxide (PBO LLINs) and pyrethroid-only LLINs without PBO (non-PBO LLINs).

### Model overview

**Fig. 1:**
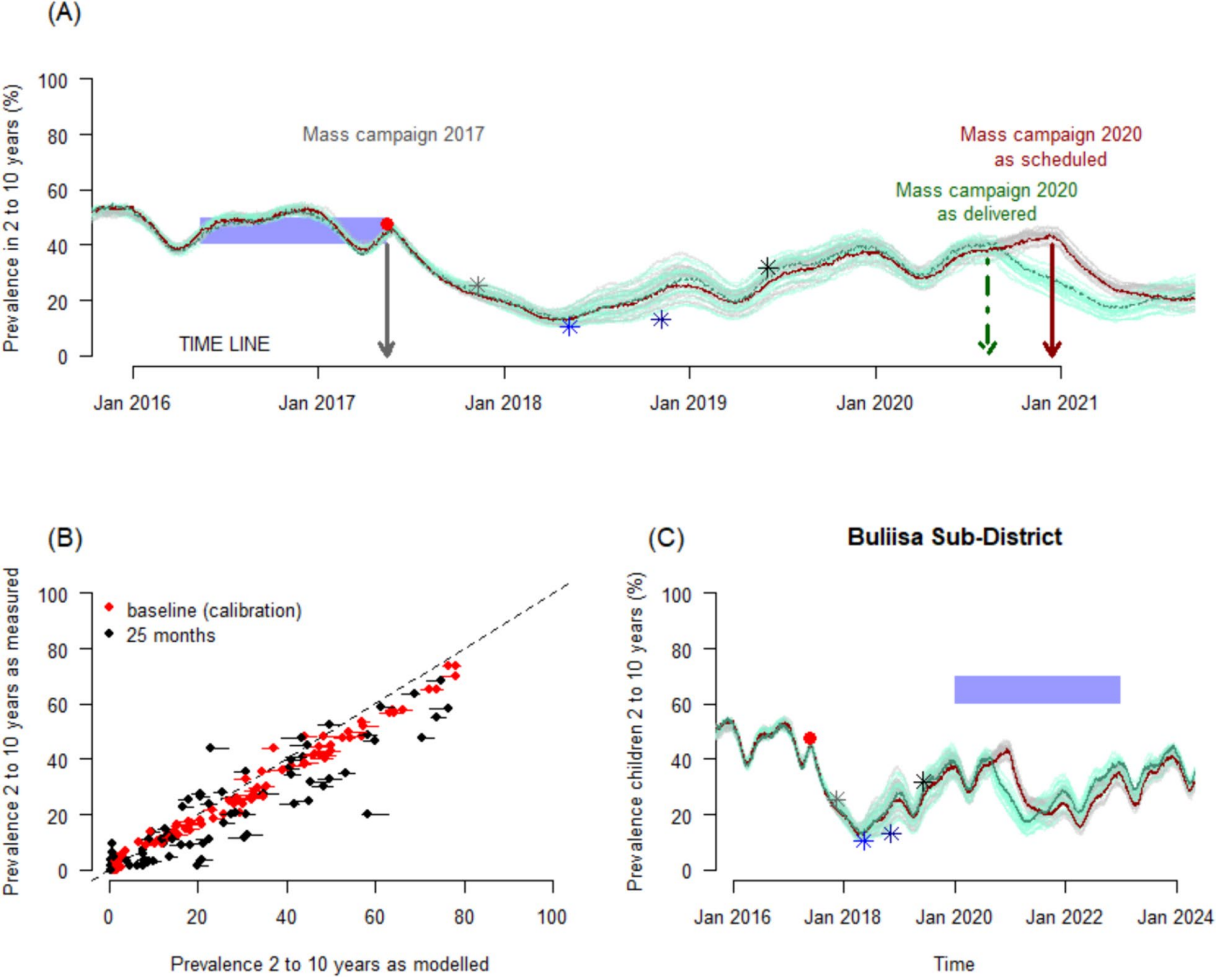
A demonstration of the model process. **A** The transmission model simulation of Buliisa sub-district. The entomological inoculation rate in the model is arbitrarily varied so that averaged annual prevalence (blue polygon) matches the empirical trial estimate measured at baseline for children aged 2–10 years (red point). In the Buliisa example, LLINs were distributed in May 2017 (first vertical grey arrow). The model simulated estimate for prevalence in the same age cohort is shown for the simulation distributing nets as originally scheduled (red line) and for the simulation distributing nets given the COVID-19 mitigation efforts (green line). Uncertainty draws, derived from varying model parameters associated with LLIN performance as determined from trial estimated data or statistical analysis of LLIN entomological impact [21], are shown as grey or green lines for the respective simulations. The mass campaign in 2020 for this location was scheduled to take place in December (dark red arrow), but took place earlier, 9th–21st August 2020 (green dot-dashed arrow). **B** Cross-sectional surveys of prevalence in children aged 2–10 years completed at baseline (red) and at 25-months (black) after the LLIN campaigns are compared to model simulated estimates for all 100 clusters in the LLINEUP trial demonstrating the model’s validity. **C** An example of the full simulation for Buliisa health sub-district. Here, the simulation is calibrated to baseline prevalence data (red point), and shown to reasonably recover the 6-month (grey), 12-month (light blue), 18-month (dark blue) and 25-month (black) empirical data estimates (Supplementary Fig. 1 shows results for all 100 clusters). Net distribution is then simulated given the scheduled date (dark red) or actual distribution date (dark green). As some distributions, like this example, went out before, and others after (see Supplementary Fig. 1), the 3-years from Jan 1st 2020 onward (blue polygon) are simulated for comparisons

An established transmission model [18] is used to simulate the trends in malaria prevalence (as measured by microscopy) from 2014 to 2023 for each of the 100 sub-districts using a transmission model for falciparum malaria [23, 24]. This process necessitates us estimating parameters from the trial data. The parameters for the transmission model are taken directly from the data collected during the LLINEUP trial wherever possible (detailed below) and otherwise default estimates are used (Supplementary Data). The transmission model is used to ensure the scenario simulated best-reflects what is understood to have happened during the trial years. To calibrate the model, the number of mosquitoes per person was arbitrarily adjusted so that simulated malaria prevalence in children aged 2–10 years old, averaged across the previous year, matched that observed in the same age group during baseline measurements (Fig. 1A) for each study cluster (health sub-district) (Fig. 1B, red points), prior to the 2017 mass campaign [16]. Each sub-district was parameterized to reflect understanding of local mosquito bionomics, net use and levels of pyrethroid resistance in local mosquito populations. Model predictions of the parasite prevalence in children age 2–10 years could then be compared visually and statistically to malaria prevalence measured empirically during the LLINEUP trial at 6, 12, 18 months (in 104 clusters) and at 25 months (in 90 clusters, 14 clusters lacked 25-month data due to COVID-19 restrictions) [22] (Supplementary Fig. 1). To do this statistically, linear regressions were fitted to each of the 4 cross-sectional surveys using the model simulated prevalence as a predictor of the empirical data estimate recorded. The absolute difference between the modelled output and empirical data were also compared to zero at each cross-sectional survey across clusters.

For the 2020–2021 campaign, the model simulated the delivery of LLINs at the (pre-pandemic) scheduled date, and then repeated this process but simulated net campaigns to match the actual delivery dates. The timing of LLIN distribution was adjusted due to challenges driven by COVID-19 mitigation measures [25]. The modelled malaria prevalence for the 2 to 10-year-old age-group (Fig. 1C) and all-age clinical incidence was estimated across the 3-years from January 2020–December 2022. Given that population size estimates for each sub-district varied, clinical incidence and the total number of cases were calculated for each sub-district, to understand whether there was any change in the benefit of nets resulting from the COVID-19 pandemic.

### The falciparum transmission model

Mechanistic models enable us to explore, whilst holding explicit assumptions, differences in potential intervention effects. In this case, the timing of intervention distributions is compared, contrasting what is expected given the actual LLINs delivery times to that given the timing of the scheduled delivery. All other parameters are matched for comparable simulations. A *falciparum* malaria transmission model [23, 24, 26, 27] is used that has been validated to demonstrate its capacity to simulate impacts from pyrethroid- and pyrethroid-PBO nets as measured in cluster-randomized trials [21]. The model has been described comprehensively elsewhere [28, 29], and the code is publicly available [18]. The human component of the model takes a non-spatial, stochastic, individual-based framework that captures the mechanisms driving malaria transmission between people and mosquitoes. The mosquito component is similar, but compartmental. Mosquito behaviours play a large role in the outcomes simulated by the model that are driven by nets. Keeping the compartmental framework means it is possible to compare ‘like-with-like’ when exploring how the timing of net distributions could change the expectation of the protection provided by LLINs, essentially reducing the stochasticity in model simulations (which can be generated using the individual-based model for the human population), helping interpretation of the results as differences restricted to the timing of LLIN distributions, or stochasticity from assumptions around human behaviours. In the model, individual humans are susceptible at birth to *Plasmodium falciparum* infection, given some level of maternal immunity which subsequently decays over the first 6-months of a person’s life [30]. The rate of exposure to infectious mosquito bites depends on local human and mosquito ecology, and the prevalence of parasites in mosquitoes [24, 27]. As humans age, their risk of developing infection is assumed to decline due to the development of acquired immunity, following continual exposure in endemic areas. Exposed humans are susceptible to clinical and severe disease, which can lead to death [24]. The transmission model was parameterized to the cluster data from the LLINEUP trial [16, 17, 19, 22, 31–33], and calibrated to baseline estimates of prevalence in children, as described below.

### Mosquito vector model

The mosquito vector model employed here is a deterministic, compartmental model for adult mosquitoes [27]. Mosquitoes hatch from eggs then progress through early and late larval stages, and develop into pupae. Only female adult mosquitoes are explicitly tracked as adults, and are assumed susceptible to malaria infection on emergence. Adult females are assumed to pursue gonotrophic cycles, whereby they host-seek (to blood-feed) and then search for aquatic habitats in which to oviposit. In the model, adult female mosquitoes are assumed to die at a constant rate, in the absence of interventions. The host-seeking, ovipositing and rate of death are affected by the presence of mosquito nets [23]. The efficacy of these nets depends on the level of pyrethroid resistance in local mosquitoes and the type of net; that is, whether it was a conventional, pyrethroid-only, or a PBO LLIN [5, 20, 21].

### Seasonality of transmission

Uganda is thought to have perennial rainfall and malaria transmission, although there is some temporal variation in both across the different sub-regions. A Fourier function with 3 cycles was fitted to sub-unit 1 level (district level) rainfall data from years 2015–2019, following Garske et al*.* [34], and noted in Griffin et al. [24]. In the mechanistic model, it is assumed that a 35-day lag in mosquito densities and, subsequently, trends in malaria incidence (peaking about 1 month after peaks in mosquito density) given this functional form.

### Mosquito species composition

Mosquito species composition is important given the different behaviours expected across species. The effects are included within the modelling framework by assuming species-specific parameters that are represented depending on the ratio of each species present in the respective clusters. For the purposes of modelling the efficacy of LLINs, experimental hut data are used to estimate statistically the average effects that induce mortality or repellence behaviours in mosquitoes [20]. There is currently insufficient data to reasonably differentiate species-specific efficacy of the different mosquito nets [20]. Therefore, it is assumed that the entomological efficacy is equivalent across all species, should a mosquito encounter a house with a net present. In the model, however, mosquito species can exhibit different propensities to feed indoors or on humans. As a result, the effect of the nets may vary with species composition. In the trial data, the primary and dominant mosquito species complex present was *Anopheles gambiae *sensu lato (s.l.) [32], unpublished estimates of species composition indicated that *An. gambiae *sensu stricto (s.s.), *Anopheles arabiensis* and *Anopheles funestus* were present. Where available, trial data are used to parameterize the model; in clusters without this information, the average proportion of each species is used (Supplementary data). In the modelling exercise, the following estimates for mosquito bionomics are assumed for each species respectively: proportion of species feeding on humans (*An. gambiae* s.s., 0.92; *An. arabiensis* 0.71; *An. funestus* 0.94), proportion of mosquitoes feeding on people in bed (0.85, 0.80, 0.78) [23, 35]. In the transmission model, the gonotrophic cycle is defined using the blood feeding rate (the regularity of feeds on humans) assuming that this happens every 3 days, resulting in a rate per day of 0.33, and the foraging time (the duration of host-seeking behaviour), which is assumed to be 0.69 days following Griffin et al*.* [23]. This is key for estimating the probability of surviving one feeding cycle and how this survival is influenced by the presence of vector control interventions to define the death rate.

### Pyrethroid resistance in local mosquitoes

Lynd et al*.* [32], observed that in Uganda there is near-universal presence of the *Vgsc-L1014F/S* marker in *An. gambiae* s.s., which infers resistance to pyrethroids (and DDT). The wild-type marker is nearly universal in *An. arabiensis.* Metabolic resistance in *An. gambiae* s.s., inferred by *Cyp4j5*-L43F (a non-synonymous change in the P450 gene) and *Coeae1d* (change in the carboxylesterase gene)*,* is also relatively high with slightly higher median estimates of metabolic resistance in the Olyset Net and Olyset Plus arms of the trial relative to PermaNet 2.0 or PermaNet 3.0 trial arms.

There is not yet sufficient understanding of the association between genotypic information and the phenotypic measures of pyrethroid resistance required for the modelling exercise. Although phenotypic measures of insecticide resistance are available from Uganda [36], data from all the different sub-districts are lacking. Therefore, estimates of the current level of pyrethroid resistance are used, derived from a publicly available database of discriminating dose WHO susceptibility bioassay tests [37]. Mean bioassay mortality were estimated at the district level for 2014, 2017 and 2020, across Uganda. The efficacy of the mass mosquito net campaign was assumed to depend on the class of net deployed, as PBO LLINs are expected to work slightly better than conventional LLINs, at most levels of resistance [38]. Information on the type of net distributed at each sub-region was used, although any potential difference between different products within the same net class are currently ignored. The level of resistance, as determined by the discriminating dose bioassay, was used to estimate the average efficacy of pyrethroid-only LLINs, following methods outlined in Nash et al*.* [20], and including pyrethroid-PBO LLINs following an updated parameterization recently presented in Sherrard-Smith et al*.* [21]. Uncertainty was carried through from the statistical analyses for model parameters estimating mosquito net efficacy [20, 21].

### Net use

**Fig. 2:**
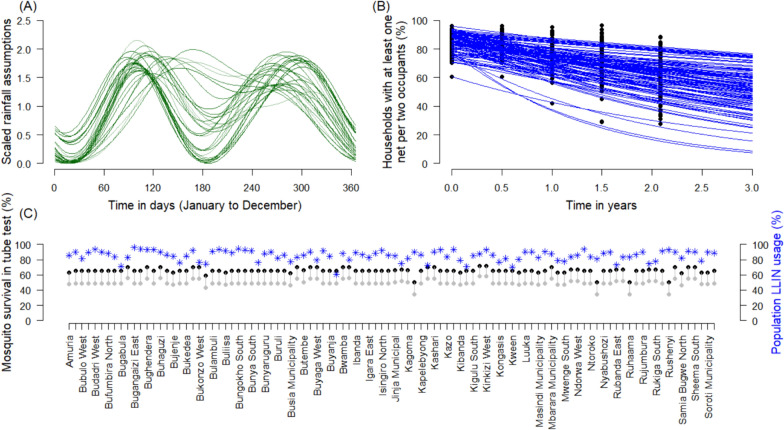
Variability in model parameters between simulated clusters. **A** Assumed and scaled seasonal profiles that determine mosquito densities in model simulations for each of 100 clusters in the model simulations. **B** Change in net use over time since the start of the mass campaign for each simulated cluster (sub-district noted alphabetically). Net use tends to drop over time, though the rate varies across sub-regions, as determined via cross-sectional surveys. Points indicate survey data from the trial (percentage of affirmative responses to question: were nets used the previous night). These data were used to fit a constant rate of net loss over time (Eq. 1, solid lines) for each health sub-district. The same net use patterns were assumed for the 2020–2021 campaign, for the respective sub-districts. **C** Model assumptions for the pyrethroid resistance (approximated as the proportion of surviving mosquitoes when tested in a discriminating dose tube bioassay exposing mosquitoes to pyrethroid insecticide) as of 2014 (grey points) and 2017 onward (black points). Assuming LLIN coverage immediately after the mass campaign (blue asterisk, right side axis)

Mass net distribution, 3-years prior to the 2017 net distributions, is simulated and the proportion of nets in use is checked to match those recorded for each sub-district. In 2017, it was assumed that trial nets were distributed instantaneously on a specified date (as actually occurred, or as planned for the original pre-pandemic schedule) and that nets were then immediately used by the recipient household replacing any older nets previously present, at the usage level recorded in the trial. No nets were assumed to be received through routine continual distribution, throughout the modelled post-pandemic period. For each sub-district, it is assumed the same initial net use and waning net use, for the subsequent 2020–2021 campaign as was achieved in this location during the 2017 efforts. The proportion of people sleeping under a net the previous night (net use) was recorded for each sub-region from the 6-, 12-, 18- and 25-months, during the LLINEUP trial (Fig. 2). A constant decay function was fitted to these data for each sub-region (Eq. 1),

1 $${Usage}_{i}={e}^{-{\sigma }_{i}t}$$

where σ is a parameter determining the rate at which people stop using nets over time *t*, following the mass campaign in Health sub-region *I* of the trial. Credible intervals for each fit were included, using 50% of the range in posterior draws, given the wide uncertainty estimated and the fact that this approach fits a single time series per cluster.

The proportion of clinically symptomatic malaria cases treated with artemisinin-based combination therapy (ACT) was fixed before and after the trial at 41%, with those receiving monotherapy at 14%. In the model, it is assumed that treatment with ACT clears gametocytes and provides a longer window of post-treatment prophylaxis than treatment with non-ACT drugs. No anti-malarial drug resistance was assumed in the parasites. Cluster-specific data were not known for clinical treatment, so it is assumed that this was constant across clusters.

### Uncertainty

Twenty uncertainty draws for parameters associated with the impact from nets were generated. The three model parameters defining; (i) induced mortality at net distribution; (ii) expected repeating behaviour at net distribution, and; (iii) the half-life of these impacts which depicts how the ITN protection wanes over time—vary with the level of pyrethroid resistance [5, 20, 21]. The posterior distribution of statistical fits to the meta-analysis of either pyrethroid-only LLINs, or pyrethroid-PBO LLINs were determined as needed for each cluster. A random binomial distribution was used to generate uncertainty in the ITN net use estimates at baseline 2017, and for 2020. The adherence to using nets (Fig. 2) was also varied using a normal distribution around the mean fits for each cluster with standard deviation 1.

## Results

### Performance of the model

A demonstration of the model process is provided in Fig. 1. Overall, the models depict a decline in prevalence of malaria among children aged 2–10 years, just after net delivery (first vertical arrow, Fig. 1A) in 2017–2018 across all health sub-districts (Supplementary Fig. 1). It is assumed that pyrethroid-only LLINs (PermaNet 2.0 and Olyset Net) were equivalent, and that pyrethroid-PBO LLINs (PermaNet 3.0 and Olyset Plus) were also equivalent, in terms of their performance at a given level of pyrethroid resistance [5, 20]. Statistically, comparing the empirical prevalence in children aged 2–10 years to the model simulated prevalence in the same age group at 6-months (linear regression adj-R^2^ = 0.87,gradient = 0.48 where 1 indicates exact agreement, degrees of freedom = 99), at 12- (adj-R^2^ = 0.82, gradient = 0.52, df = 99), 18- (adj-R^2^ = 0.85, gradient = 0.54, df = 99), and 25-months (adj-R^2^ = 0.92, gradient = 0.79, df = 86) after the 2017–18 mass distribution campaign, provides some validation of the model process. While the model underestimates the measured impact early on (simulating higher prevalence estimates for the 6–18-month period), it captures the 25-month data (68% of clusters are matched within 10-percentage points)—the most recent estimate of burden—reasonably well (Fig. 1B). The underestimations at earlier time points are likely due to mismatching the seasonality profile in the clusters for the trial year (or seasonal behaviour of mosquito vectors), perhaps mischaracterizing pyrethroid resistance in the model compared to locally, or indicates the method underestimates initial impacts from LLINs more generally. Given that the aim was to assess the likely impact of shifting LLIN deployment due to COVID-19 interruptions, any mis-matches here will carry through for both scenarios allowing the analysis to continue. Nevertheless, this is recognized as a limitation. It was also assessed whether clusters that received LLINs earlier than originally scheduled during the 2020–21 campaign were simulated equivalently well to those receiving nets later than scheduled by fitting regressions to the cross-sectional surveys with a binary predictor for early or late nets. There were no substantial differences (Supplementary Fig. 2) indicating the model simulations should be reasonable moving forward for the purposes of the addressed question.

### Effect of delayed LLIN delivery on malaria burden

**Fig. 3:**
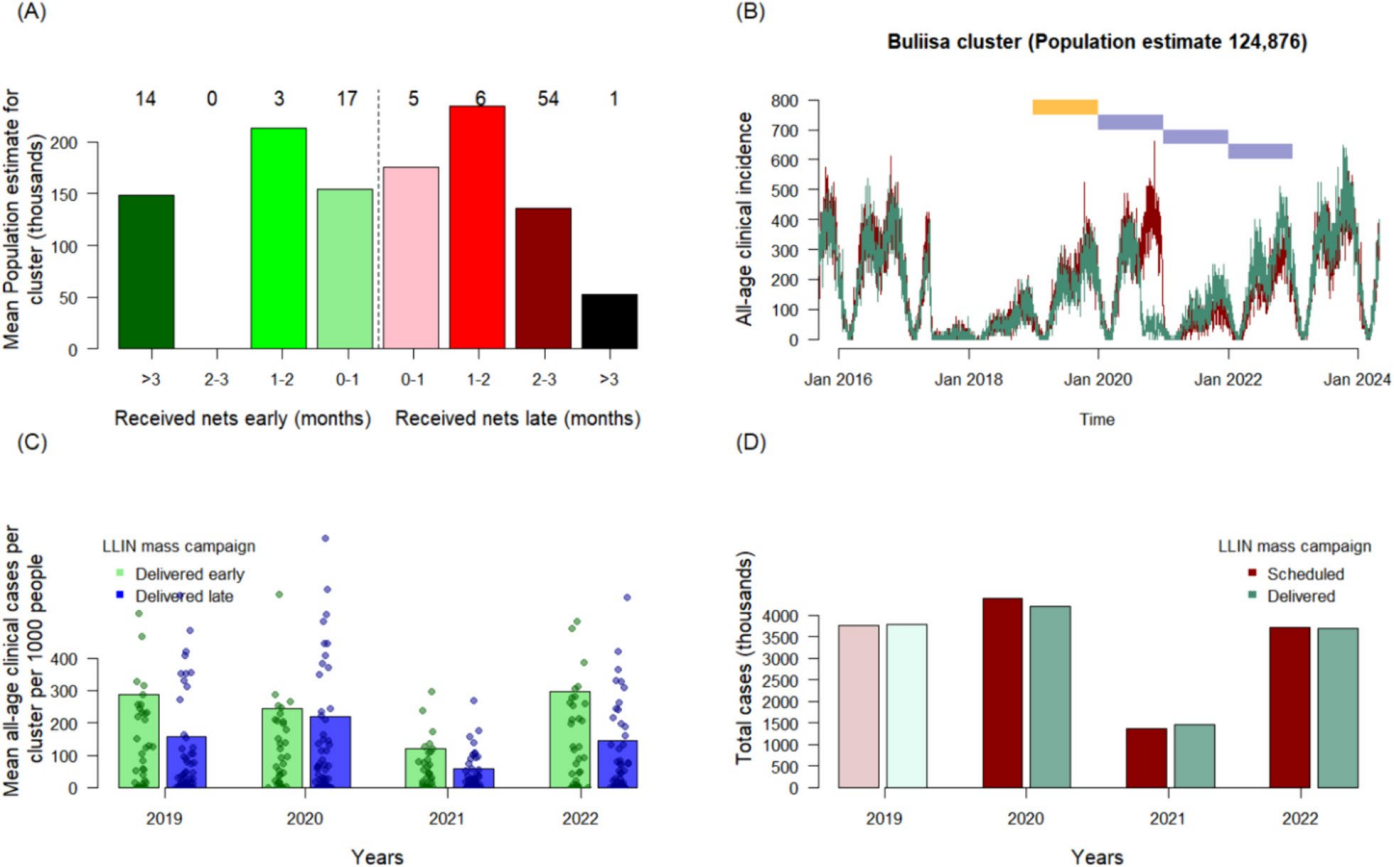
Modelled estimates of the impact on malaria of changing the timing of the mass LLIN campaign in 2020–21 caused by the COVID-19 pandemic. In **A**, the population size in each sub-district varied, with most people residing in the sub-districts where net distributions were delayed compared to those distributed early relative to the schedule prior to interruptions from the pandemic (numbers of sub-districts in each category shown). The transmission model simulated all-age clinical incidence daily so that it was possible to compare 2019–2023 impacts given the scheduled, or delivered mass campaign. **B** Illustrates this for the Buliisa health sub-district which was scheduled to receive LLINs in Nov-Dec 2020 (red simulation), but received the nets in August 2020 (green). The polygons mark the years for comparison. In **C**, the model-estimated clinical cases per 1,000 people were calculated across the 4 years from January 2019 for clusters (individual points), according to whether nets were delivered before or after the scheduled time. **D** Shows the estimated total number of cases in the region (thousands of cases) for each year, given the schedule or the actual campaign dates across the 100 clusters

Most people (approximately 64% of the recipient population, across 66 of the 100 clusters) received LLINs later than scheduled in 2020–21, although some clusters received LLINs over 3 months earlier than planned (Fig. 3a). Using the same example cluster as Figs. 1, 3b demonstrates the model simulated, all-age, clinical incidence estimates for the two scenarios in Buliisa where the mass campaign in 2020 was scheduled to take place in December, but was actually delivered earlier, from 9th–21st August 2020. The summed clinical incidence projected in 2019–2023 was compared (Fig. 3c).

By chance, on average, clusters that received LLINs earlier than planned had a higher incidence of malaria cases in 2019, prior to the 2020–21 mass distribution campaign (Fig. 3c). Taking into consideration the timing of the LLIN distribution, seasonality in transmission, the waning efficacy of nets, and their loss of use over time, the model indicates that those sub-districts that received LLINs late had substantially higher incidence of cases in 2020. This is likely because of the assumptions for this analysis on the seasonal transmission of malaria in Uganda. This is evident in the example (Fig. 3b): in the model, it is assume that malaria parasite transmission will be lower in February–March (due to a dip in mosquito densities) and then increase again for most of the year. This pattern is repeated every year. Nets are continually losing efficacy in the model, and net use by residents is also assumed to reduce steadily following distribution. Thus, just prior to a campaign, net efficacy and use will be at the lowest point. But in these simulations, the lowest burden of malaria is expected in the early months of the calendar year so that there are fewer cases to avert by any intervention. In some circumstances, early LLIN distributions might be less impactful than the originally scheduled plan (which may have seen LLINs arrive as transmission increases, so that there are many cases to avert).

On average, simulations predict broadly similar mean incidence of cases in 2021 and 2022. Once differences in cluster population size and the timing of the LLIN distributions were taken into consideration, the model predicts no substantial increase in malaria burden caused by COVID-19 mitigation measures, with similar numbers of malaria cases predicted whether the mass campaign happened as scheduled (total cases predicted across all 100 clusters: 4,398,500 in 2020, 1,372,000 in 2021, and 3,722,100 in 2022 rounded to nearest 100) or as the LLIN were delivered (4,212,300 in 2020, 1,468,800 in 2021, 3,679,400 in 2022) (Fig. 3d).

## Discussion

The impact of COVID-19 on global public health cannot be overstated [6]. The pandemic and subsequent restrictions imposed to limit transmission and combat COVID-19 disrupted malaria control activities across Africa, interfering with the mass distribution of long-lasting insecticidal nets and access to diagnostic testing and effective anti-malarial treatment, and raising concerns that malaria morbidity and mortality would increase markedly [1, 6, 7]. In March 2020, the government of Uganda acted quickly to enforce strict COVID-19 restrictions, disrupting the 2020–2021 universal coverage campaign (UCC) to distribute LLINs nationwide [25]. A recent analysis of Uganda’s 2020–2021 UCC found that, despite the COVID-related challenges, 27,789,044 LLINs were distributed to 11,287,392 households, with 2.5 LLINs distributed per household on average. Overall, 94% of households in Uganda received at least one net, surpassing the goal of 85% household net ownership [25]. In Uganda, there are an average 5.1 people per household [25], suggesting the country may have been able to achieve high usage, however explicit data on this metric is lacking within the current study.

The model suggests that the altered timing of the LLIN delivery by a few months during the 2020–2021 UCC did not substantially impact malaria burden in the LLINEUP study area in 2021–2022. These findings are consistent with those reported from 17 rural health facilities in Uganda during the first year of the COVID-19 pandemic showing no major effects on malaria disease burden and indicators of case management [39]. It was found that LLINs were distributed earlier than originally planned in some study areas, which may have offset the impact of delays in distribution elsewhere, explaining the lack of impact on total malaria burden. However, in some circumstances, early LLIN distribution appears to have had less of an impact than the originally planned delivery schedule due to attrition of nets prior to the peak of transmission, and reduced protection due to naturally waning efficacy of LLINs over time (both of which were assumed to match observed data for net use and efficacy from the trial). A study conducted in 12 districts across Uganda [17] found that 1–5 months post-LLIN distribution most households (93.4%) owned at least one LLIN distributed through the UCC, but only 56.8% were adequately covered by UCC LLINs (owning at least 1 UCC LLIN for every 2 residents), suggesting that the number of nets distributed to many households was insufficient.

The most recent WHO World Malaria Report indicates that the number of presumed and confirmed malaria cases nationwide, which fell from 17.5 million cases in 2020 to 15.1 million in 2021, increased by 32% to 20 million in 2022 [1]. The 2022 World Malaria Report [40] suggests that the retention of LLINs in Uganda is less than 2 years, based on work from [41] together with the expectation that the mortality inducing impact from LLINs will wane relatively rapidly because of pyrethroid resistance in vector populations, the lower estimates of cases in 2021 and slight resurgence in 2022 reported in the World Malaria Report 2023 [1] make sense, and the presented simulations are in line with these trends. Multiple factors have likely contributed to this surge in malaria cases in Uganda, including inadequate coverage with current malaria control tools, widespread insecticide resistance, emerging artemisinin resistance, dynamic vector species composition and behaviours, and inadequate funding [1]. This modelling study suggests that LLIN attrition, possibly due to delivery of an insufficient number of top-up nets after the mass campaign and limited LLIN lifespan [25, 42, 43], was potentially a more important contributing factor than the COVID-19 related disruptions in LLIN delivery. The important issues of adequate coverage and net attrition have been raised previously in Uganda [17, 22], including the need for more frequent mass campaigns (every 2 years)—particularly if the effect of PBO on pyrethroid-PBO nets wanes quickly [44]—transmission and strategies to ensure high levels of LLIN coverage during national distribution campaigns [17]. Expanding distribution of new generation LLINs, including pyrethroid-PBO LLINs and dual active ingredient LLINs, which have been shown to provide superior protection against malaria as compared to pyrethroid-only LLINs [16, 22, 44, 45], is also needed given widespread metabolic resistance of *Anopheles* vectors to pyrethroid insecticides across sub-Saharan Africa. However, the trend of reduced cases in 2021 followed by resurgence in 2022 may also be partly due to strict restrictions brought in by the Ugandan Government in 2020. These included social distancing and the suspension of public gatherings, school closures and curfews [25]. The last restriction is particularly interesting because it may have resulted in people going to bed earlier and, therefore, benefiting more from the use of an LLIN in the context of malaria transmission through 2021.

This modelling study had several limitations. First, only 90 of the 104 clusters were included in the main trial at 25 months due to COVID-19 restrictions. Fortunately, this reduction in cluster number had little impact on the power of the study. Second, all modelling analyses have limitations resulting from the explicit assumptions made by the modelling process. The full model code and assumptions have been presented previously [21]. In the present analysis, while specified for clusters, data were assumed to be consistent across health sub-districts over time given the information recorded through the trial, including net use, trends in mosquito abundance and drug treatment. It was also assumed no routine continual distribution. Some of these assumptions may not have been correct although they may not alter the inference for within-district comparison—as they would equally impact on both the counterfactual and alternative scenarios simulated. Uncertainty draws were determined around key parameters that may interact with simulated impacts from ITNs to mitigate for this. Some specific limitations that apply to the present analysis are: (i) that it was assumed net use as measured in the LLINEUP trial is consistent for each health sub-district in the following mass LLIN campaign; (ii) that mosquito abundance patterns, and corresponding peaks in seasonal malaria transmission (see Figs. 1B, 3B), are consistent across all the years simulated; (iii) that mosquito bionomics are constant and these estimates account for the overlap in activity between people and mosquitoes, so the analysis is also assuming no major changes in social behaviour associated with risk of mosquito biting; (iv) that the reduced impact from mosquito nets in the presence of mosquitoes that are able to survive exposure to pyrethroid insecticide is predictable [20, 21]; (v) that the same net type (either conventional LLIN or PBO LLIN) is deployed across the health sub-district in the 2020–2021 campaign; (vi) that drug treatment is consistent across all health sub-districts. In addition, malaria transmission is sensitive to environment and weather changes that play out year-on-year. Here, a Fourier function is assumed that repeats annually so any environmental effects that are specific to the focus year will be missed. This variation, were it explicitly modelled, would be the same in the comparable simulations, however, so would not likely impact the conclusions. The key question that was the aim to address with this analysis is how much did changing the delivery dates of the mass campaign distribution impact malaria control efforts in Uganda? Therefore, the decision was made to explicitly keep all parameters constant for each health sub-district for which data were available from the LLINEUP trial, while varying the timing of the net distributions. Consequently, with the limitations and assumptions listed, the modelling approach can reasonably serve the purpose to estimate differences in protective impact.

## Conclusions

Initial models suggested that interruptions in LLIN distribution campaigns and other health services could result in substantial increases in malaria cases and deaths [6, 7], however, evidence of this impact is mixed [1, 46]. It was found that the altered timing of LLIN delivery in 2020–21 due to stringent measures instituted by Ugandan government did not substantially impact overall malaria burden. The model suggests that a similar level of protection for the community was maintained, as would have been expected in the absence of the pandemic. In some circumstances, the model suggests that early LLIN distribution appears to have had less of an impact than the originally planned schedule. This is due to the assumptions in the model of net attrition prior to the peak of transmission, and reduced protection due to naturally waning efficacy of LLINs to induce mosquito death over time.

### Supplementary Information

Supplementary Material 1.

## Data Availability

All data generated or analysed during this study are included in this published article and its Supplementary Data file. All code is available via Supplementary Information.

## Abbreviations

COVID-19: Severe acute respiratory syndrome coronavirus 2 (SARS-CoV-2)
LLIN: Long-lasting insecticidal nets; in this context, the abbreviation is used for both pyrethroid treated nets and pyrethroid plus piperonyl-butoxide treated nets.
PBO: Piperonyl-butoxide, a synthetic pesticide used on nets together with pyrethroid insecticide that acts as a synergist to recover the mechanism of action of the active pyrethroid.
MoH: Ministry of Health
