# Supplementary material for: LLIN Evaluation in Uganda Project (LLINEUP): modelling the impact of COVID-19-related disruptions on delivery of long-lasting insecticidal nets on malaria indicators in Uganda
Source: Malar J. 2024 Jun 6;23:180. doi: 10.1186/s12936-024-05008-8 (PMC11157881; doi:10.1186/s12936-024-05008-8)

**Supplementary Material**

**LLIN Evaluation in Uganda Project (LLINEUP) – Modelling the impact of COVID-19-related disruptions on delivery of long-lasting insecticidal nets on malaria indicators in Uganda**

Jaffer Okiring^1,2*^, Samuel Gonahasa^2^, Catherine Maiteki-Sebuguzi^2^, Agaba Katureebe^2^, Irene Bagala^2^, Peter Mutungi^2^, Simon P Kigozi^2^, Jane F Namuganga^2^, Joaniter I Nankabirwa^1,2^, Moses R Kamya^2,3^, Grant Dorsey^4^, Martin J Donnelly^5,6^, Thomas S Churcher^7^, Sarah G Staedke^5,8^, Ellie Sherrard-Smith^7^

**Modelling additional information and code**

All model code is stored in the repository here:

<https://github.com/EllieSherrardSmith/uganda_covid_impact>

All data estimates and parameters for each simulation are documented in the accompanying Supplementary Data file.

**Supplementary figures**

**Figure S1** shows each simulation using median parameter draws and 20 uncertainty draws varying net impact associated parameters. Net distributions are compared from Jan 1^st^ 2020 – Dec 31^st^ 2022 (blue bar) given nets were distributed as scheduled or as actually delivered, depending on Covid-19 mitigations. The trial baseline estimates are shown as red points to which each median simulation is calibrated by arbitrarily adjusting the entomological inoculation rate within the model simulation. We use this estimate of EIR for uncertainty simulations for the respective clusters. The follow up cross-sectional surveys from the LLINEUP 1 RCT are shown as asterisks at 6-months (grey), 12-months (light blue), 18-months (dark blue) and 25 months (black) after the 2017 mass LLIN campaign for each cluster where available. The red line, with grey uncertainty lines, shows the scheduled net distribution scenarios from Jan 2020 onward. The green lines show the actual delivery as completed.


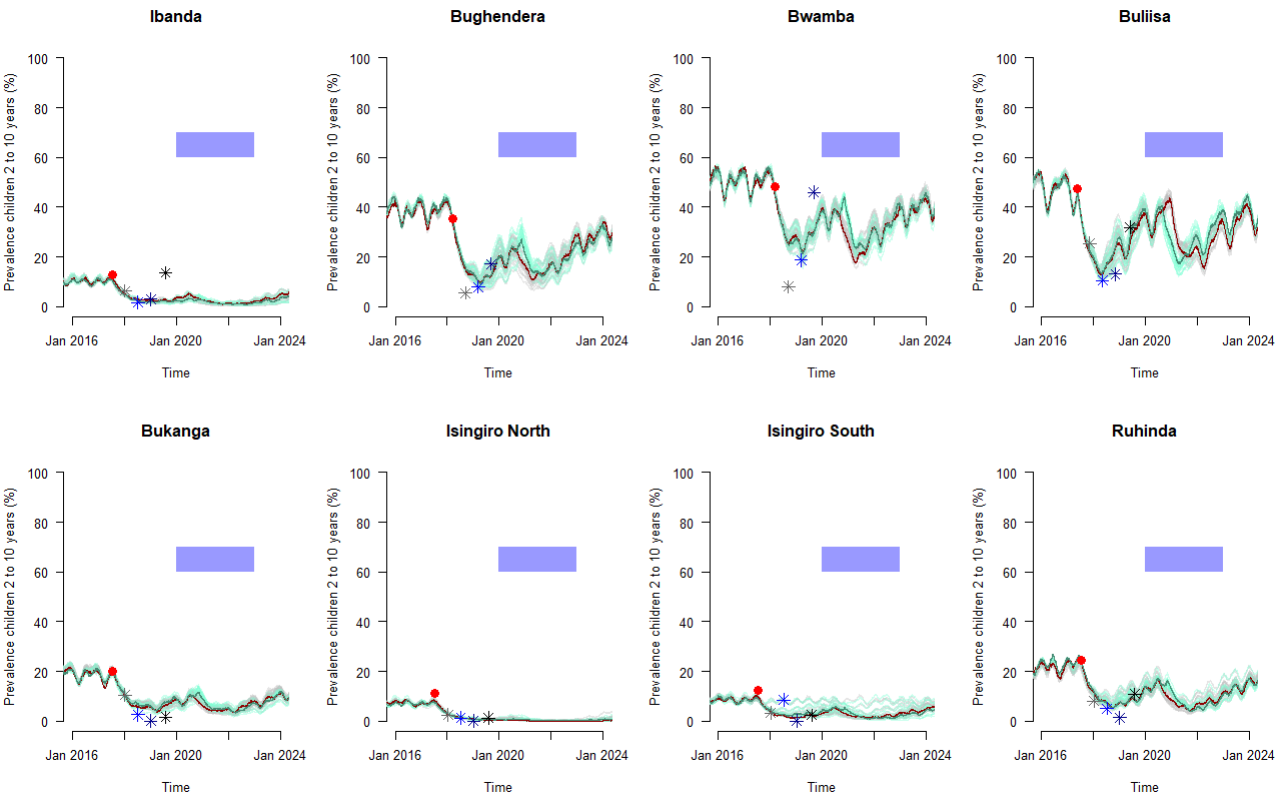


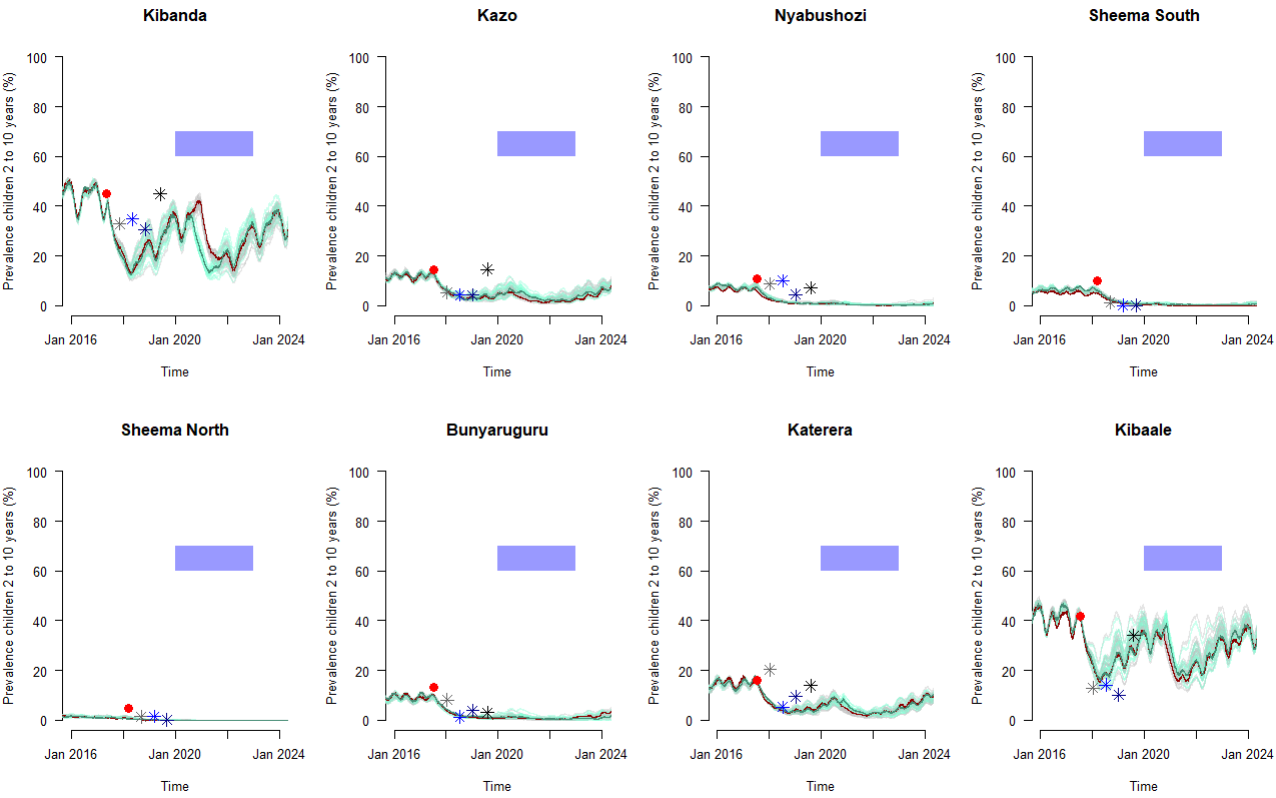


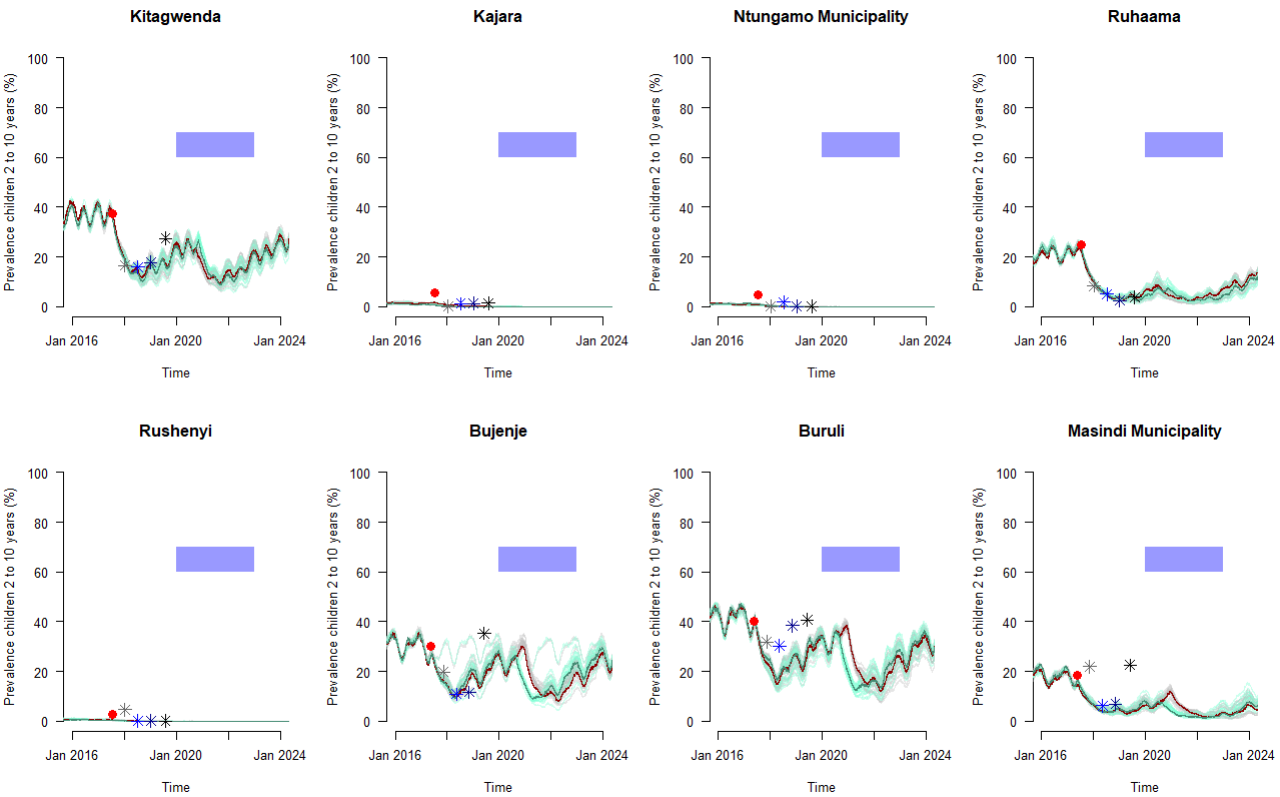


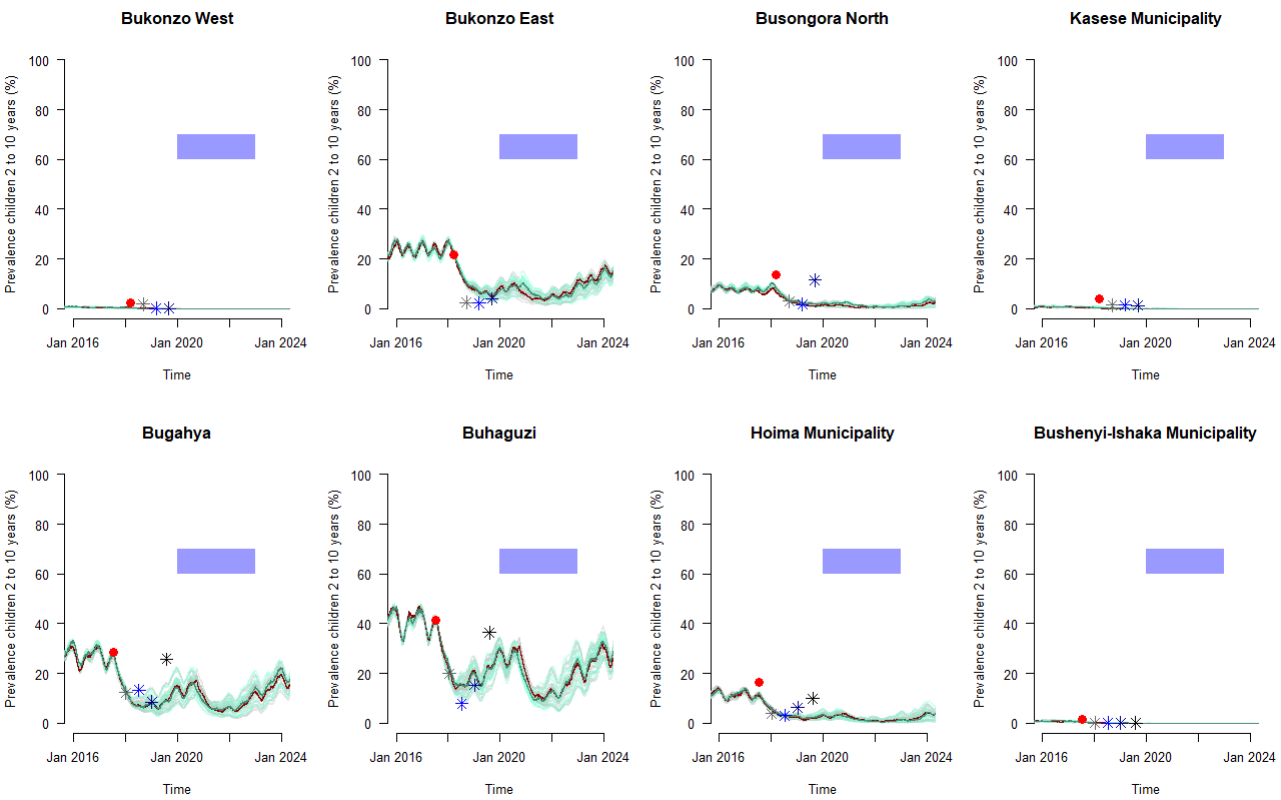


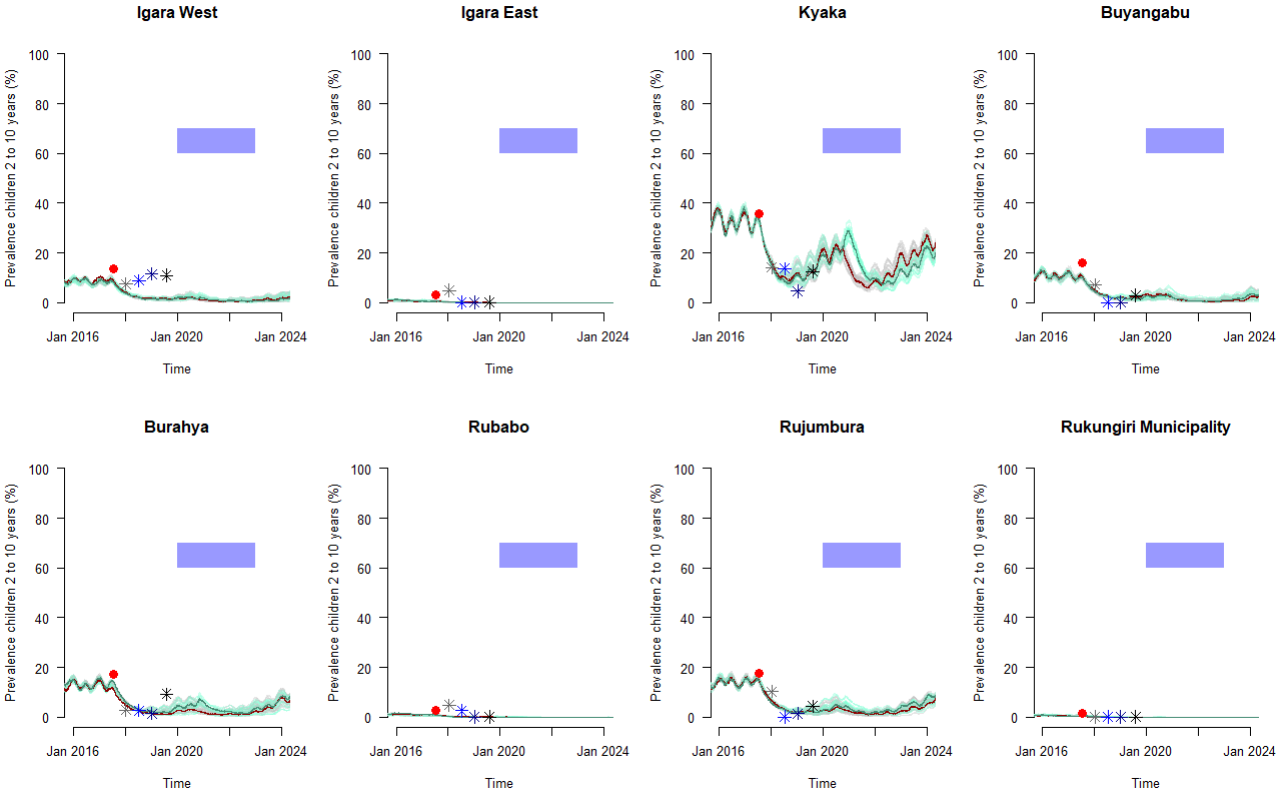


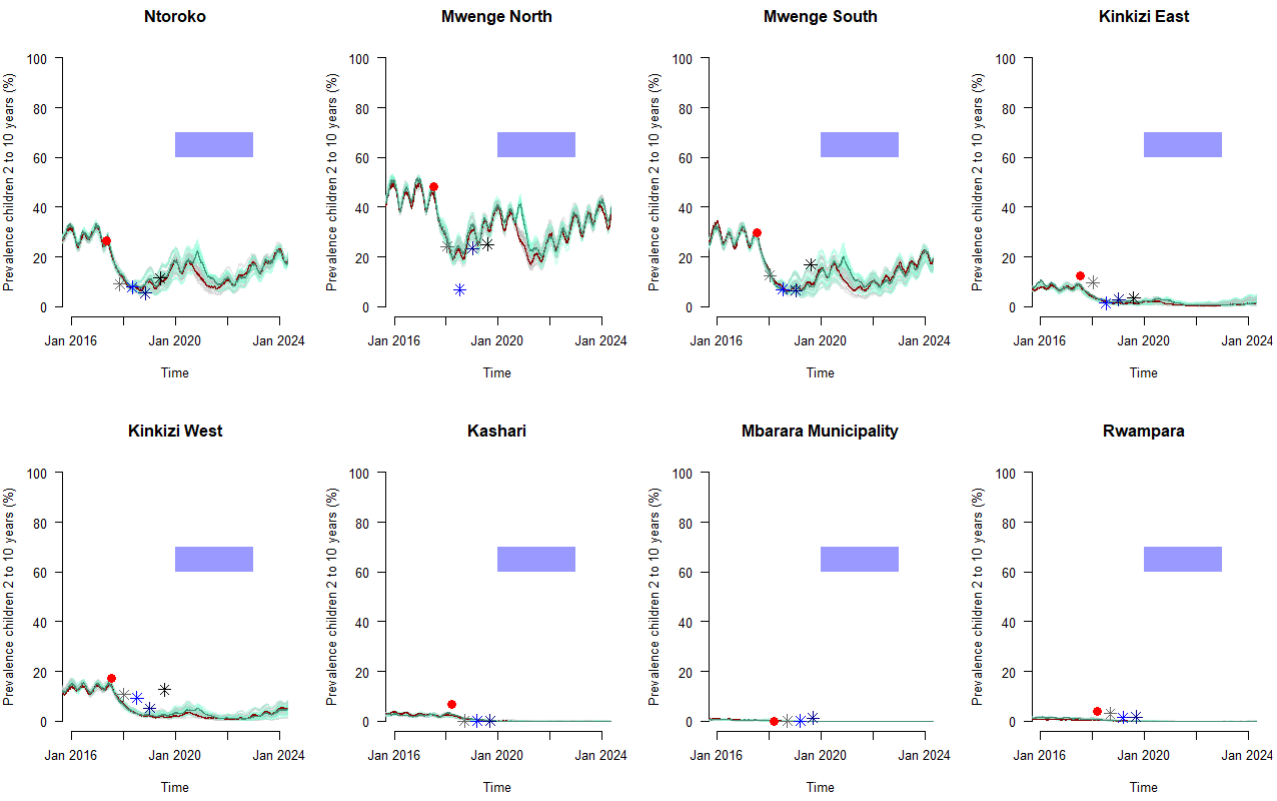


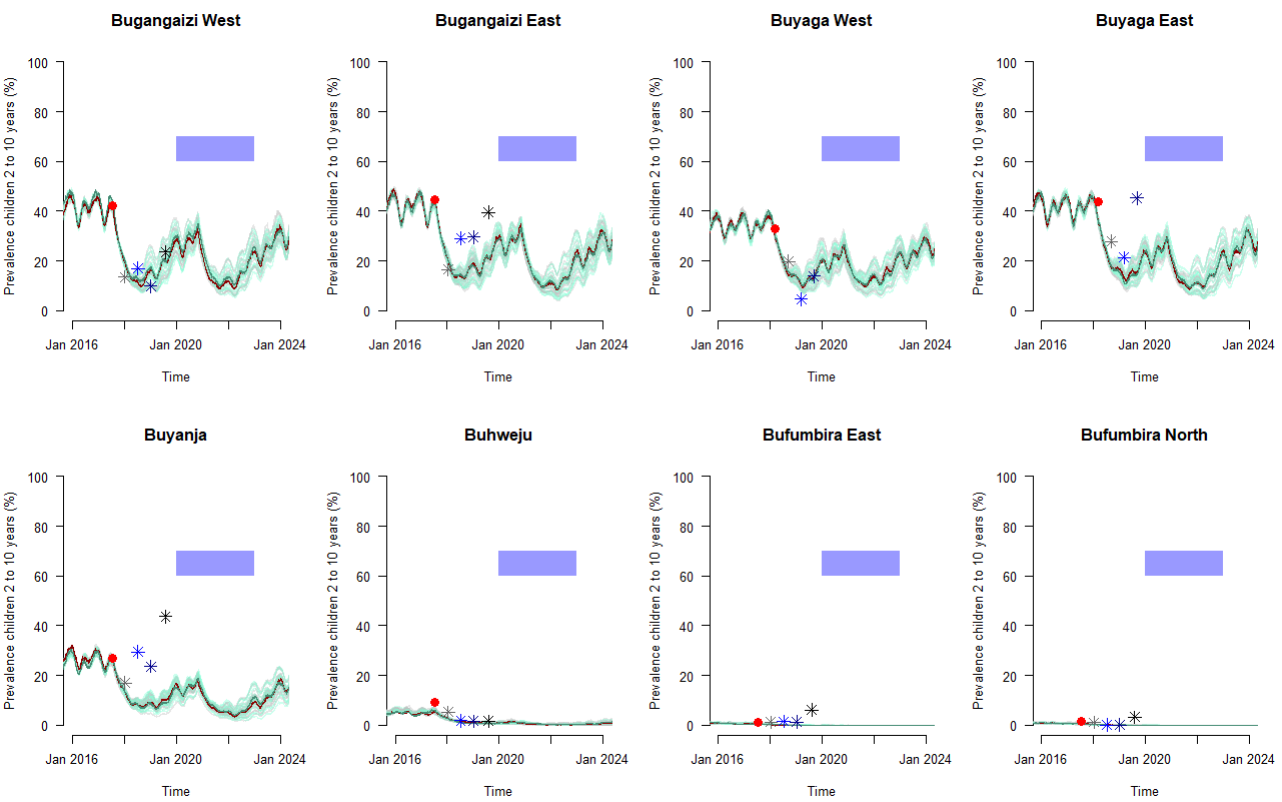


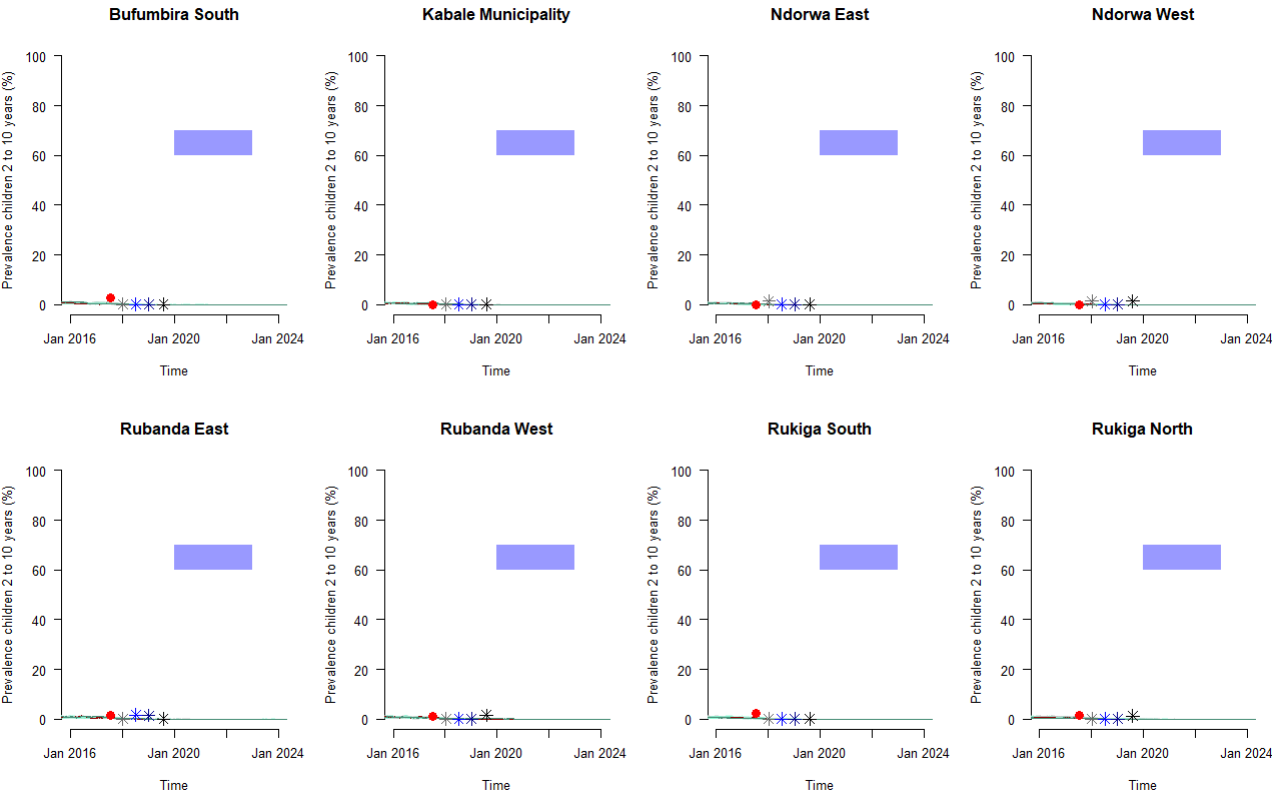


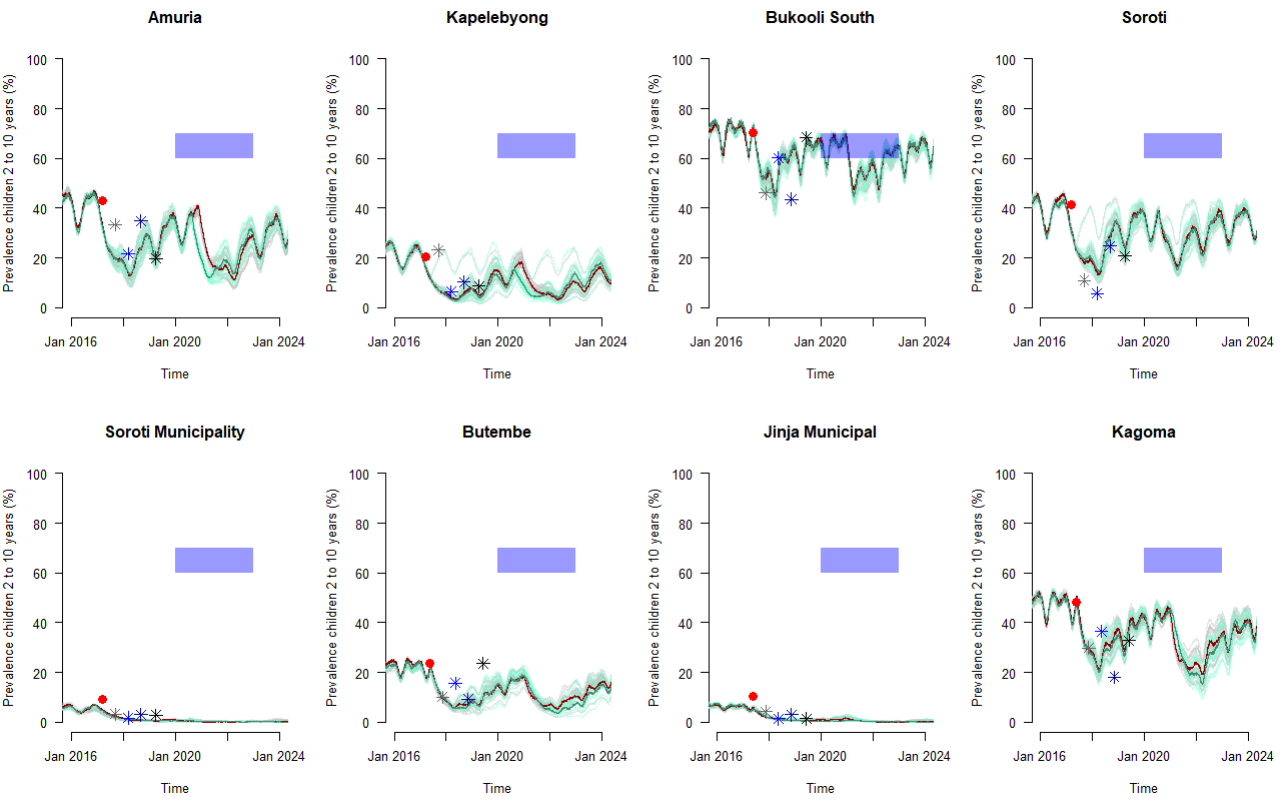


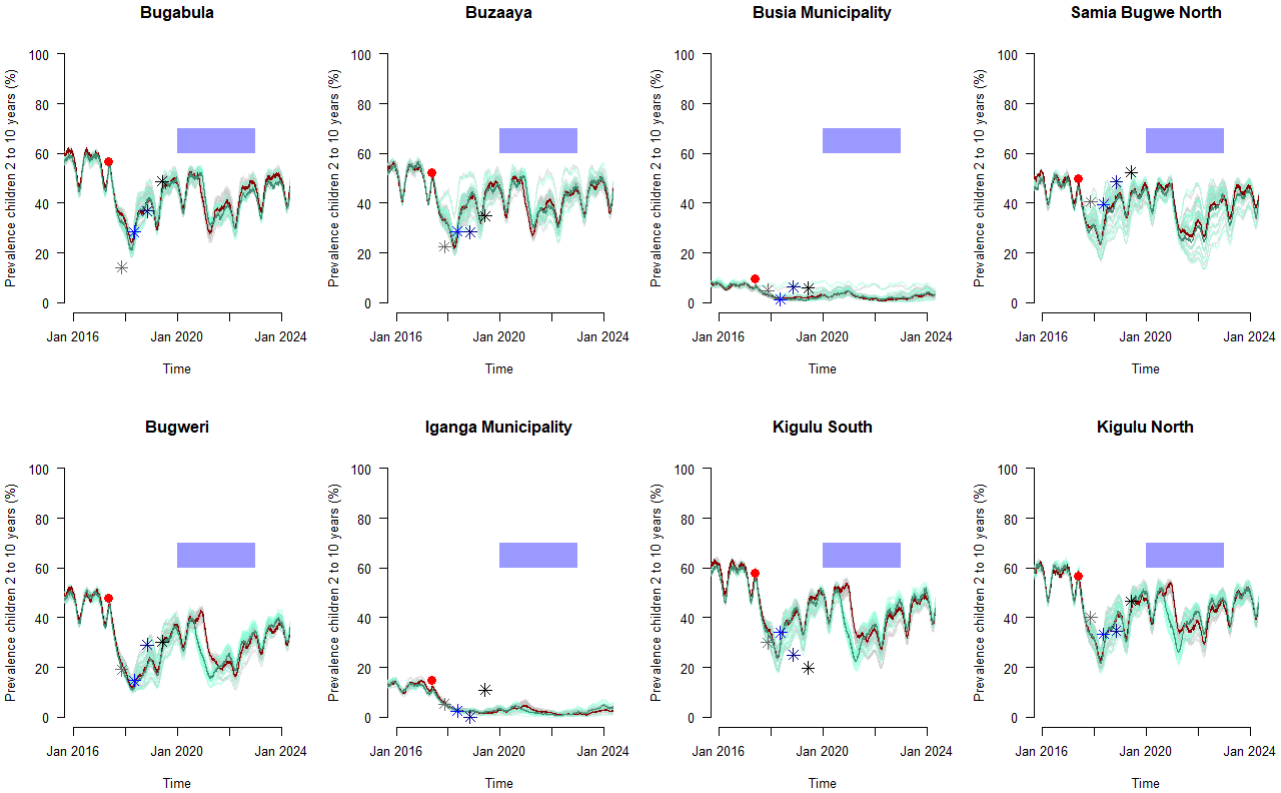


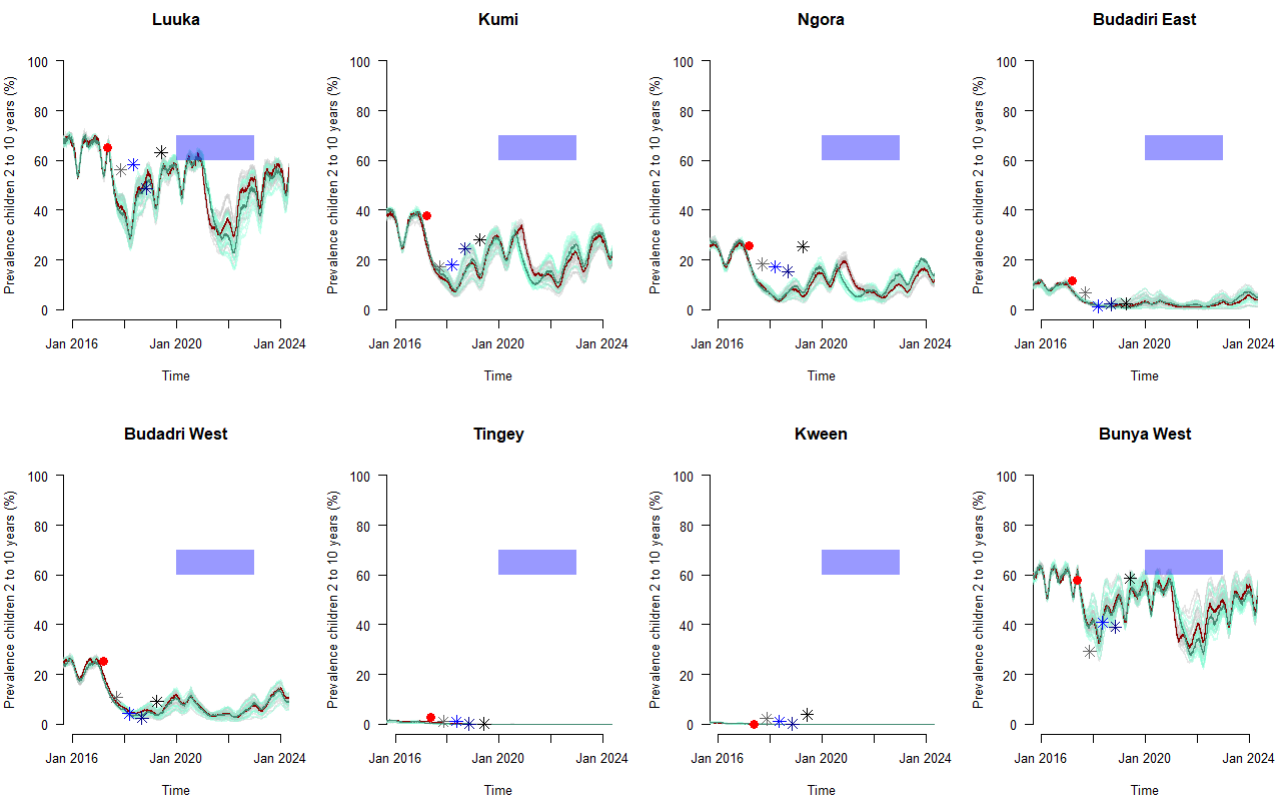


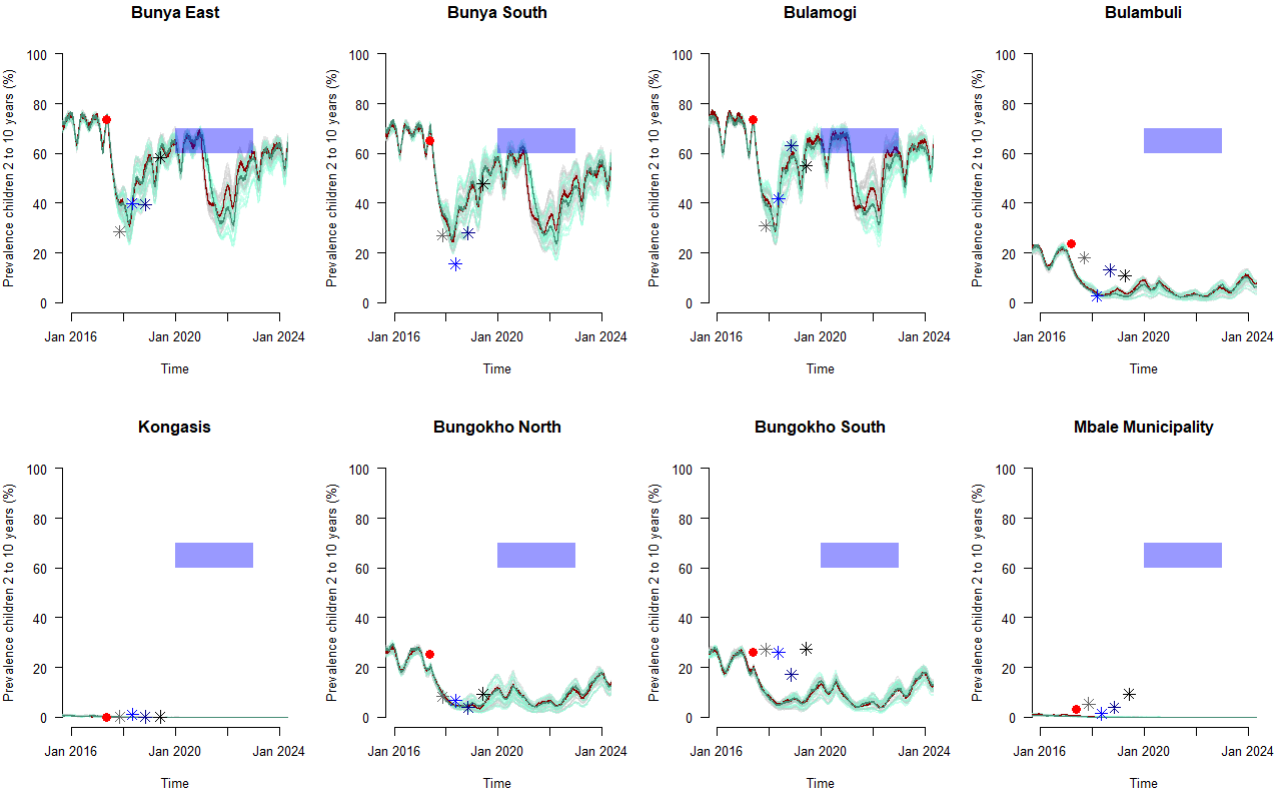


**
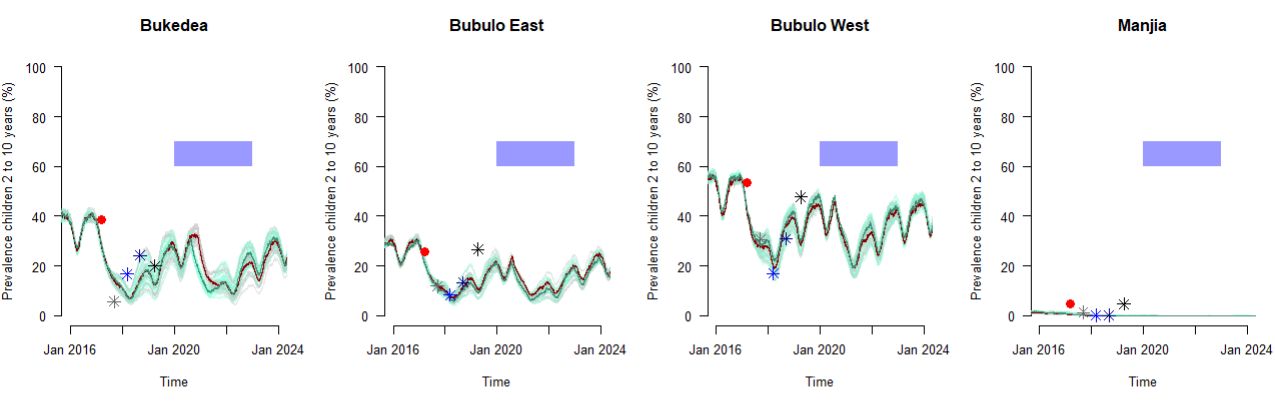
**

**Supplementary Figure 2**: Comparisons of the unity between model outcome predictions and cross-sectional surveys of prevalence in children of 2 to 10 years of age from LLINEUP 1 trial (Staedke et al. 2019). Solid points indicate clusters that received nets later than planned because of the Covid-19 pandemic, open points show those receiving net earlier than planned. The model predicts both situations similarly as indicated by the linear regressions shown as solid (later nets) or dashed (earlier nets) lines.


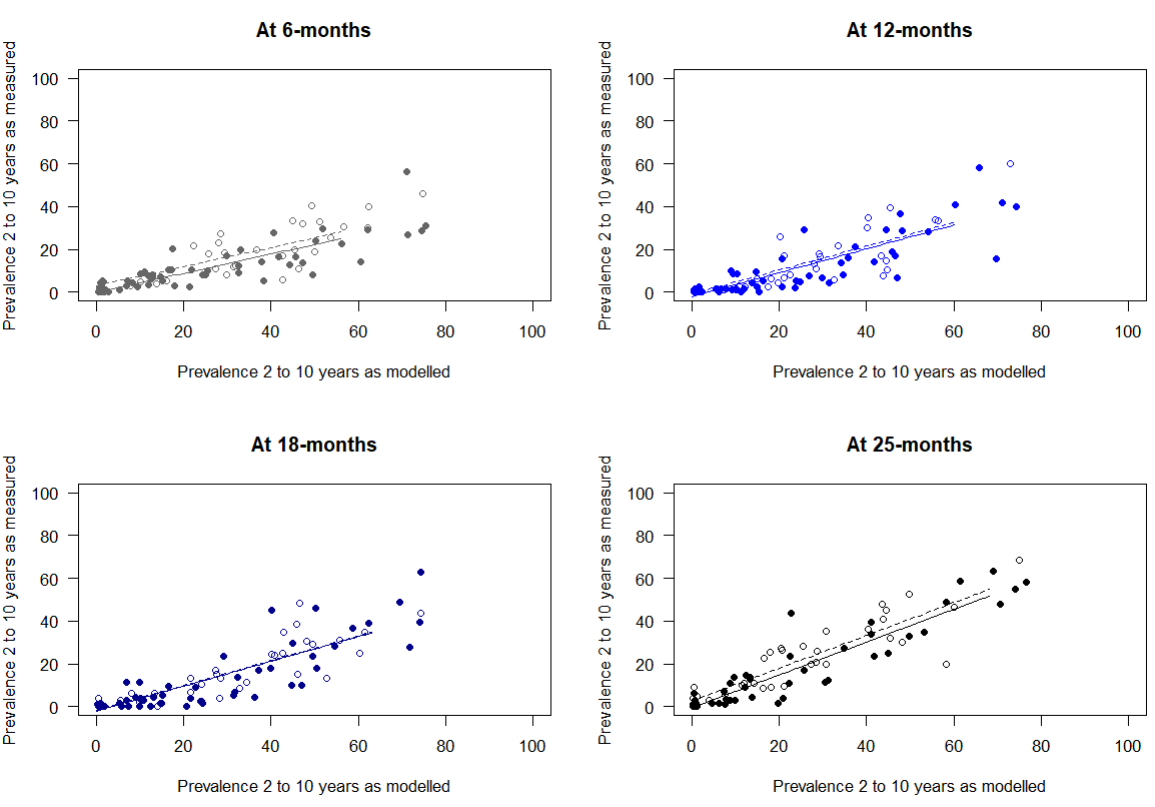

Supplement: Supplementary file 1 — Supplementary Material 1. [file 12936_2024_5008_MOESM1_ESM.docx]
